# Identification of crucial genes of pyrimidine metabolism as biomarkers for gastric cancer prognosis

**DOI:** 10.1186/s12935-021-02385-x

**Published:** 2021-12-14

**Authors:** Zhengxin Wu, Jinshui Tan, Yifan Zhuang, Mengya Zhong, Yubo Xiong, Jingsong Ma, Yan Yang, Zhi Gao, Jiabao Zhao, Zhijian Ye, Huiwen Zhou, Yuekun Zhu, Haijie Lu, Xuehui Hong

**Affiliations:** 1 School of Medicine, Guangxi University, Nanning, 530004 China; 2 Department of Hematology, The First Affiliated Hospital of Xiamen University and Institute of Hematology, School of Medicine, Xiamen University, Xiamen, 361003 China; 3 Institute of Gastrointestinal Oncology, School of Medicine, Xiamen University, Xiamen, 361000 China; 4 Department of Gastrointestinal Surgery, Zhongshan Hospital, Xiamen University, No. 201-209 Hubin South Road, Xiamen, 361004 Fujian China; 5 Organ Transplantation Institute of Xiamen University, Fujian Provincial Key Laboratory of Organ and Tissue Regeneration, School of Medicine, Xiamen University, Xiang An South Road, Xiamen, 361102 China; 6 National Center for International Research of Biological Targeting Diagnosis and Therapy, Guangxi Medical University, Nanning, 530000 China; 7 Department of Colorectal Surgery, The First Affiliated Hospital of Harbin Medical University, Harbin, 150001 Heilongjiang China; 8 Department of Radiation Oncology, Affiliated Zhongshan Hospital of Xiamen University, Xiamen, 361102 China

**Keywords:** Pyrimidine metabolism, GC, Bioinformatics, Prognosis risk model, Biomarker

## Abstract

**Background:**

Metabolic reprogramming has been reported in various kinds of cancers and is related to clinical prognosis, but the prognostic role of pyrimidine metabolism in gastric cancer (GC) remains unclear.

**Methods:**

Here, we employed DEG analysis to detect the differentially expressed genes (DEGs) in pyrimidine metabolic signaling pathway and used univariate Cox analysis, Lasso-penalizes Cox regression analysis, Kaplan–Meier survival analysis, univariate and multivariate Cox regression analysis to explore their prognostic roles in GC. The DEGs were experimentally validated in GC cells and clinical samples by quantitative real-time PCR.

**Results:**

Through DEG analysis, we found NT5E, DPYS and UPP1 these three genes are highly expressed in GC. This conclusion has also been verified in GC cells and clinical samples. A prognostic risk model was established according to these three DEGs by Univariate Cox analysis and Lasso-penalizes Cox regression analysis. Kaplan–Meier survival analysis suggested that patient cohorts with high risk score undertook a lower overall survival rate than those with low risk score. Stratified survival analysis, Univariate and multivariate Cox regression analysis of this model confirmed that it is a reliable and independent clinical factor. Therefore, we made nomograms to visually depict the survival rate of GC patients according to some important clinical factors including our risk model.

**Conclusion:**

In a word, our research found that pyrimidine metabolism is dysregulated in GC and established a prognostic model of GC based on genes differentially expressed in pyrimidine metabolism.

**Supplementary Information:**

The online version contains supplementary material available at 10.1186/s12935-021-02385-x.

## Introduction

Gastric cancer (GC) remains one of the most common malignant diseases in the world [1, 2]. Although the treatment has made some progress over the decades, the 5-year survival rate of patients with advanced GC remains low [3]. Exploration and analysis of tumor prognostic biomarkers are crucial for assessing tumor progression, predicting the effect of treatment, reducing recurrence and mortality, and prolonging survival.

Metabolic reprogramming is one of the characteristics of cancer, promotes tumor cell proliferation and survival [4, 5]. A lot of studies have shown that the metabolism of sugar, lipid and amino acid ultimately affects tumor growth through nucleotide metabolism [6–10]. Nucleotide metabolism is a multi-step process containing a variety of enzymes, including common catalytic enzymes and rate-limiting enzymes such as lyase, synthase, amidotransferase, dehydrogenase, etc. Studies have also proved that restraining the activity of some rate-limiting enzymes in pyrimidine metabolism can directly affect tumor growth [11, 12]. For example, high expression levels of rate–limiting enzymes carbamoyl-phosphate synthetase 2, aspartate transcarbamylase, and dihydroorotase (CAD), deoxythymidylate kinase, 5ʹ-nucleotidase, cytosolic II (NT5C2), NT5C3, ribonucleotide reductase catalytic subunit M1 (RRM1), RRM2, thymidine kinase 1 (TK1), TK2, dihydroorotate dehydrogenase (DHODH), thymidylate synthetase, uridine-cytidine kinase 2 (UCK2), UCKL1 in pyrimidine metabolism are described in liver cancer and lung cancer patients and related to poor clinical prognosis [11, 13]. Using pyrimidine metabolism rate-limiting enzymes CAD and DHODH as targets to inhibit pyrimidine synthesis enhances the molecular therapeutic response to glioblastoma [14]. 5ʹ-nucleotidase ecto (NT5E) is related to poor clinical prognosis and regulates cell proliferation and migration in many cancers including GC [15, 16]. Historically, pyrimidine nucleotide synthesis has been the pathway of choice to target tumors, because pyrimidine nucleotides are the fundamental building block of DNA synthesis in cells and are increasingly needed by cancer cells due to its rapid growth [12]. Pyrimidine analogue 5-Fluorouracil (5FU) is one of the most extensively used drugs in cancer treatment. 5FU can inhibit thymidylate synthase and prevent the conversion of deoxyuridine acid to thymidylate, thus interfering with DNA synthesis [17]. It is commonly employed to treat breast, colorectal, pancreatic, gastric, liver, and ovarian cancer [18]. However, pyrimidine analogues like 5FU not only target the pyrimidine metabolism of tumor cells, but also partially affect the pyrimidine metabolism of normal cells, causing great side effects [19]. Thus, it is of great significance to search for genes differentially expressed in pyrimidine metabolism according to different cancer types for the treatment and prognosis of different cancer.

**Fig. 1:**
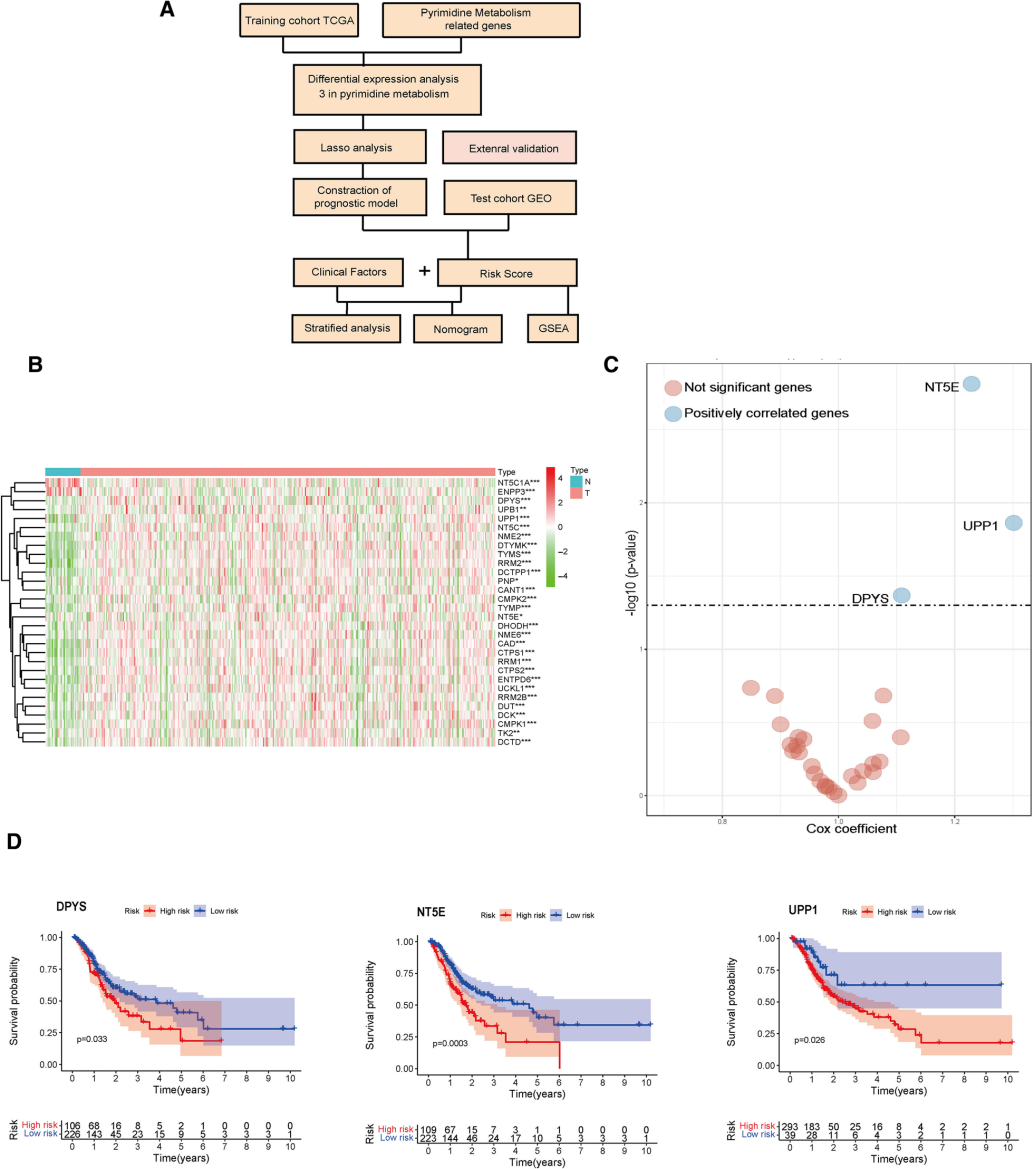
Differential gene expression analysis in the TCGA database. **A** Flow chart of the study. **B** Heatmap of differential gene expression in pyrimidine metabolism. **C** Unicox analyses of DEGs according to prognosis. **D** Kaplan–Meier survival curves of OS for these 3 genes in TCGA

In this research, we employed The Cancer Genome Atlas (TCGA) cohort to explore the differentially expressed genes (DEGs) in pyrimidine metabolism in GC and verified them through in vitro experiments. A prognostic risk models was established based on these DEGs. Stratified survival analysis, univariate and multivariate Cox analysis of this model confirmed that it is a reliable and independent clinical factor. Therefore, we made nomograms to visually depict the survival rate of GC patients according to some important clinical factors including our risk model. These conclusions have been verified in the Gene Expression Omnibus (GEO) database. The detailed workflow chart of our article was shown in Fig. 1A.

## Methods

### Acquisition of information of GC patients

The training cohort of mRNA expression information and relevant clinical data of 375 cases of GC patients (10 cases without survival information and 33 cases with survival time less than 30 days were eliminated later) and 32 cases of normal people were all downloaded from TCGA (https://www.cancer.gov/), which were expressed as fragments per kilobase million (FPKM). Finally, 332 samples were included in the study. Common clinical characteristics including age, sex, stage, TNM grade, clinical survival time, and clinical survival outcome were included in our analysis. The test cohort of mRNA gene expression data expressed as FPKM were downloaded from GSE15459 and GSE84433 cohort in GEO (https://www.ncbi.nlm.nih.gov/geo/), which includes 615 cases of GC patients. The clinical features including age, gender and stage were download from their original paper [20, 21] 0.57 pyrimidine metabolism pathway genes (map00240) were employed in KEGG (https://www.kegg.jp).

### Construction of correlation analysis and protein–protein interaction (PPI) network

After obtaining the data of DEGs in GC from TCGA, in order to analyze the relationship between these genes, we did correlation analysis employed R packages "ggstatsplot" and "corrplot", Pearson's correlation coefficients were used to analyze the correlation of the three genes. Using GeneMANIA database to process genes that co-expressed with NT5E/UPP1/DPYS in GC samples and established protein–protein interaction analysis.

### Establishment and verification of a prognostic risk model

The Wilcoxon method was utilized for DEGs analysis, and then the pheatmap software package of R software v1.2.1 was employed to draw the heat map. Univariate Cox analysis was performed on overall survival (OS) to screen DEGs with prognostic values. Lasso-penalized Cox regression analysis was utilized to remove redundant genes with low impact, and a prognostic risk model was established on basis of the expression of residual DEGs mRNA. The obtained prognostic risk model was subsequently verified by the GSE15459 and GSE84433 cohorts in GEO. We use the survminer package in the R program to test the capability of the model, and use the surv_cutpoint function to calculate the optimal cut-off value. According to this value, GC patients were divided into high risk cohort and low risk cohort. Subsequently, time-dependent receiver operating characteristic (ROC) curves were plotted with time ROC package to estimate the predictive power of the prognostic model. In order to assess the difference in overall survival (OS) between the high-risk cohort and the low-risk cohort, stratified Kaplan–Meier survival analysis was utilized.

### Cox regression analysis

Univariate and multivariate Cox regression analysis were employed to test whether the prognostic risk model was an important and independent clinical factor. *p* < 0.05 was considered as statistically significant.

### Gene set enrichment analyses (GSEA)

In order to explore whether GC pyrimidine metabolism disorders will affect other signaling pathways, we performed GSEA analysis on the high-risk and low-risk cohorts in the GEO and TCGA databases, respectively. GSEAv4.1.0 tool was employed to combine with the KEGG gene set for GSEA analysis. *p* < 0.05 and FDR < 0.25 were considered as statistically significant.

### Establishment and validation of a predictive nomogram

We established a nomogram model to evaluate the OS prognosis of GC patients via employing the rms package of R program. The coxph function of survival package was used to find the C index, which was used to measure the prediction ability and performance of the model. Then, the lrtest function of rms package was used to measure the advantages of each model.

### Cell culture

One normal human gastric epithelial cell GES-1 and six GC cell lines MKN-28, MKN-45, MGC-803, HGC-27, BGC-823 and SGC-7901 were collected from the Affiliated Zhongshan Hospital of Xiamen University. GES-1, MKN-28 and MKN-45 were cultured in RPMI 1640 medium (Gibco, USA), MGC-803, HGC-27 and BGC-823 were cultured in Dulbecco’s Modified Eagle’s medium (Gibco, USA), and SGC-7901 was culture in Minimum Essential Medium (Gibco, USA) in a humid environment with 5% CO2 and 37 °C. All types of media contained 100 U/mL penicillin–streptomycin solution (Meilun Biotech, Dalian) and 10% fetal bovine serum (Gibco, USA).

### Human GC samples

20 pairs of GC tissues and matching normal tissues (At least 5 cm or farthest from the tumor) were collected from the Department of Gastrointestinal Surgery, Affiliated Zhongshan Hospital of Xiamen University.

### RNA extraction and quantitative real-time (q-RT)PCR

Following instructions provided by the manufacturer, TRIzol reagent (TransGen Biotech, Beijing) was utilized to extract RNA from cells and tissues. The cDNA Synthesis Supermix kit (TransGen Biotech, Beijing) was utilized to reverse transcribe 1 µg RNA into cDNA. 2X SYBR Green qPCR Master Mix (Bimake, USA) was utilized to carry out real-time PCR on a BioRad Biosystems 7500 instrument (Bio-Rad, Hercules, CA) in triplicate. β-actin or 18 s-rRNA was employed to normalize the levels of RNA measured. All operations should be done on ice as much as possible. The sequences of the primers are as follows: β-actin-F: GGACTTCGAGCAAGAGATG and β-actin-R: AGCACTGTGTTGGCGTACAG; 18 s-rRNA-F: AGTCCCTGCCCTTTGTACACA and 18 s-rRNA-R: GATCCGAGGGCCTCACTAAAC; NT5E-F: TCTTCTAAACAGCAGCATTCC and NT5E-R: CATTTCATCCGTGTGTCTCAG; UPP1-F: ACTGCCCAGGTAGAGACTATC and UPP1-R: CTGCACCAGCTTCTTGTTAAG; DPYS-F: ACCCGACTTCCTCATGAATCT and DPYS-R: CATCCGATCTTCAACACCATTCA.

### Western blot analysis

GC cells were laid in 10 cm culture dishes and cultured to 75% to 90% confluency and harvested in lysis buffer containing proteases and phosphatase inhibitors, leave it at 4 °C for about 45 min. The protein was quantified by BCA analysis. Then the proteins were isolated on SDS-PAGE and then transferred onto polyvinylidene difluoride (PVDF) membranes, these membranes were probed using primary antibodies and secondary antibodies according to the supplier's recommendations: DPYS (1:500, Proteintech, 13,237–1-AP, Wuhan, Hubei), NT5E (1:1000, Abcam, ab133582, Suite Cambridge, USA), UPP1 (1:1000, Abcam, ab128854, Suite Cambridge, USA), β-actin (1:1000, #3700, CST, USA), anti-rabbit secondary antibody (Abcam, ab150077, Cambridge, USA) and anti-mouse secondary antibodies (Bio-Rad, 1706516, Hercules, CA). Finally, enhanced chemiluminescence (ECL) was used to observe the results.

### Immunohistochemistry

Briefly, the collected tissues were fixed, embedded, and mounted on slides. After deparaffinization and rehydration were finished, the antigen was repaired by gastric enzyme (Maxim, DIG-3009, Fuzhou, Fujian). Later, an immunohistochemical UltraSensitive Sp kit (Maxim, KIT-9730, Fuzhou, Fujian) was used to suppress the endogenous peroxidase activity in tissues and block the sections. The indicated antibody was applied in the cold room overnight. The corresponding secondary antibody was used on the next day. Finally, an Enhanced DAB chromogenic kit (Maxim, DAB-2032, Fuzhou, Fujian) was used to achieve the detection. Hematoxylin and Hydrochloric acid ethanol were used to stain and treat the slides which were then mounted and observed under microscopy.

## Results

### DEGs related to GC pyrimidine metabolism in TCGA

**Fig. 2:**
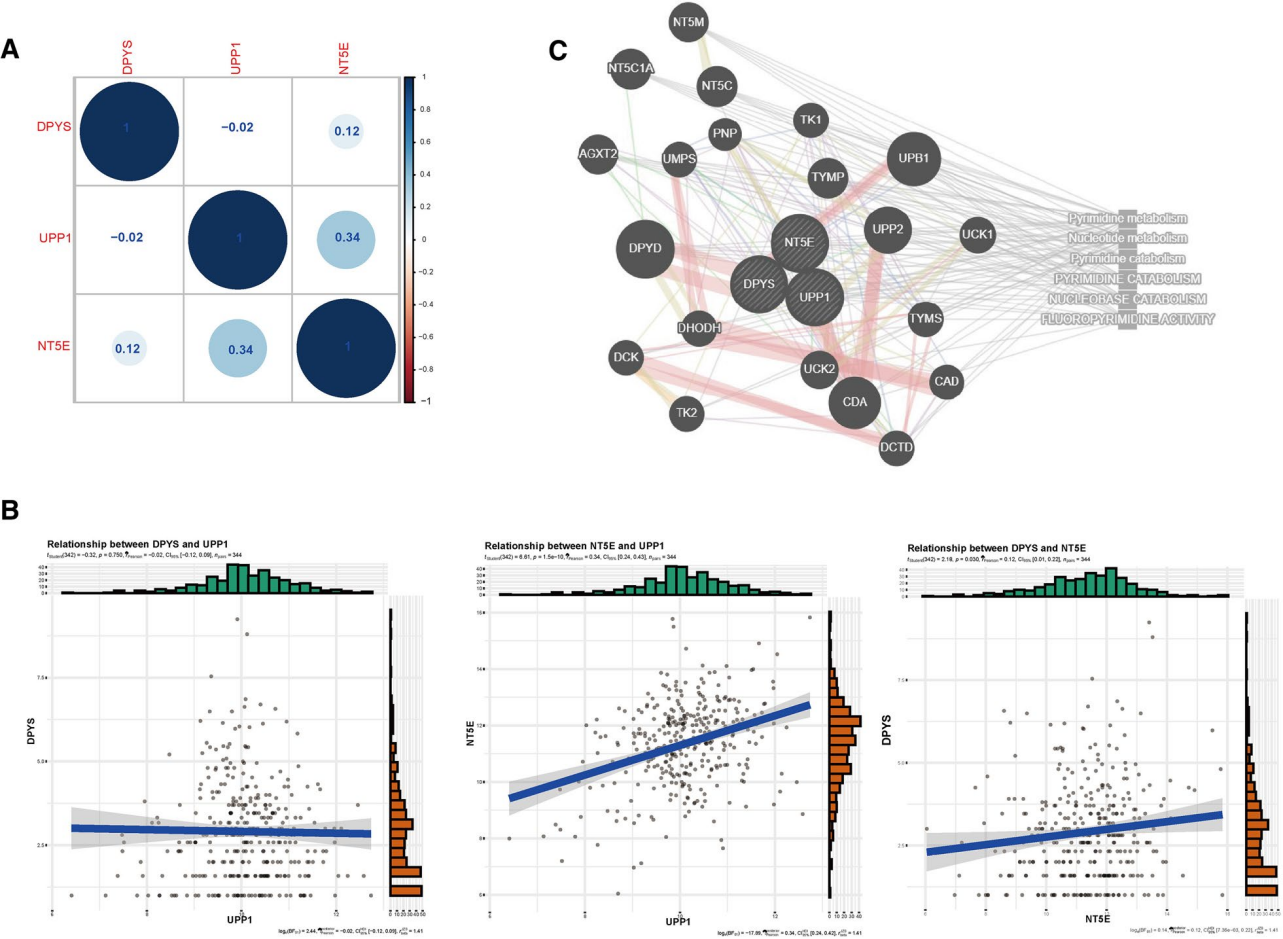
The mRNA expression correlation of these DEGs and PPI network in GeneMANIA database. **A** Correlation plot of NT5E, DPYS and UPP1. **B** Pearson's correlation analysis of expression levels of NT5E, DPYS and UPP1. **C** PPI network between NT5E, DPYS and UPP1 with their co-expression genes

To seek DEGs in GC pyrimidine metabolism, we compared mRNA expressions in 332 GC tissues and 32 normal gastric tissues in TCGA. Through univariate Cox regression analysis, 3 genes (NT5E, DPYS and UPP1) connected with pyrimidine metabolism were detected (Fig. 1B, C). Survival analysis and Lasso-penalized Cox analysis showed that these three genes in GC pyrimidine metabolism were closely connected with GC prognosis (Fig. 1D, Additional file 1: Fig. S1). Among the 3 genes related to pyrimidine metabolism, NT5E and dihydropyrimidinase (DPYS) participate in the catabolism of pyrimidine, and uridine phosphorylase 1(UPP1) is an enzyme in the salvage synthesis pathway of pyrimidine nucleotides. We then analyzed the associations between these three genes. We found that NT5E is positively correlated with DPYS and UPP1 UPP1(r = 0.12, *p* = 0.03; r = 0.34, *p* = 1.5E-10, respectively), while the DPYS is negatively correlated with UPP1(r = − 0.12, *p* = 0.75) (Fig. 2A, B). To further analyze the functional correlation of the three genes, we established a PPI network by employing GeneMINEA database. These three key genes and their co-expressed genes are mainly involved in fluorouracil activation and promotion of pyrimidine metabolism (Fig. 2C).

### Experimental validation of DEGs in GC cells and tissues

**Fig. 3:**
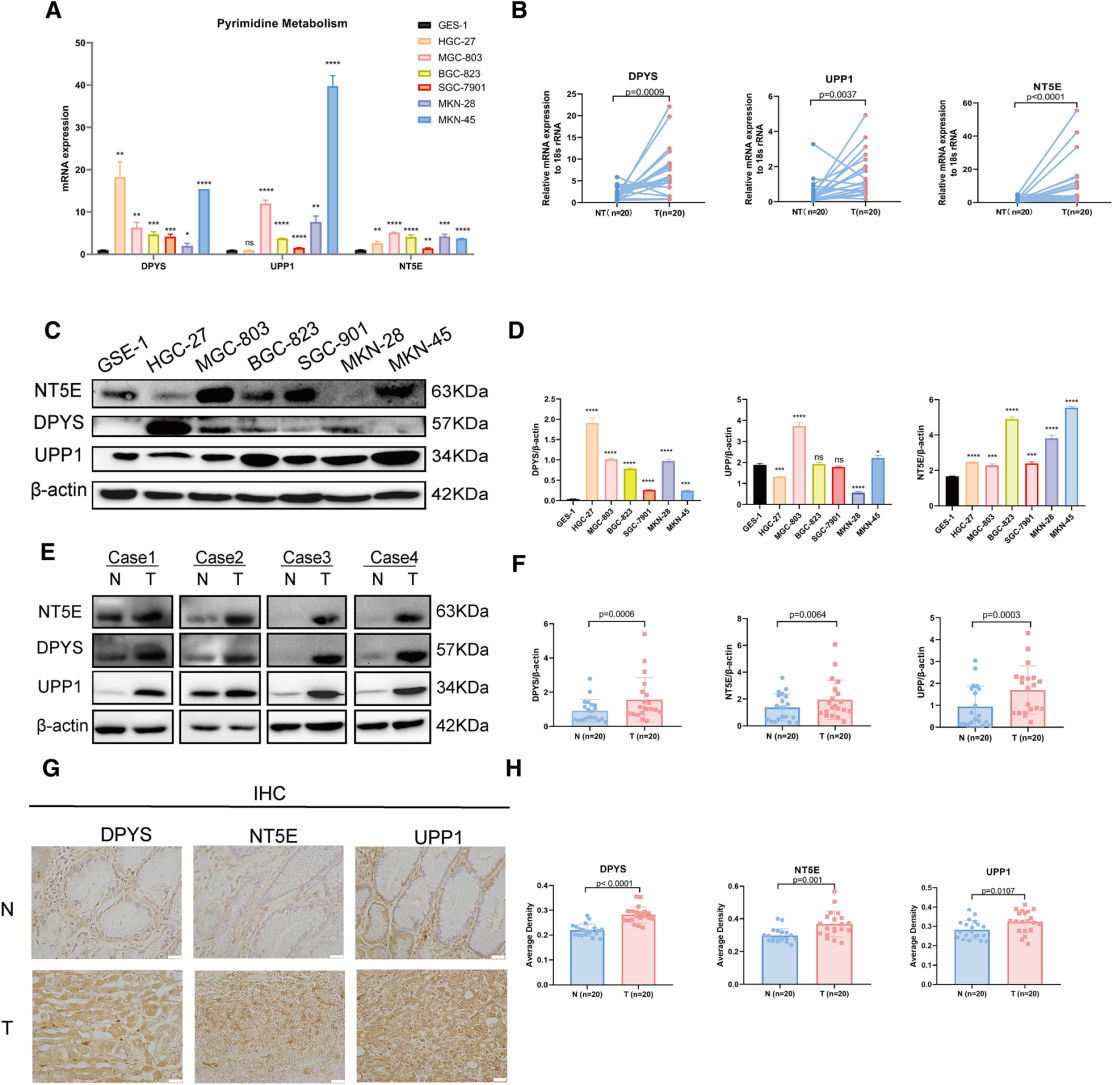
The DEGs in pyrimidine metabolism are overexpressed in GC. **A** The mRNA expressions of pyrimidine metabolism (NT5E,UPP1 and DPYS) were accessed in six GC cell lines (including HGC-27, MGC-803, SGC-7901, BGC-823, MKN-45 and MKN-28) and one immortalized normal gastric epithelial cell GES-1. **B** The mRNA expressions of pyrimidine metabolism were also compared between 20 pairs of GC tumor tissues and adjacent non-tumor gastric tissues. **C**, **D** The expression of NT5E,UPP1 and DPYS were analyzed by western blot between normal gastric cell and GC cell lines. **E**–**H** The expression of NT5E, UPP1 and DPYS was analyzed by western blot and immunochemistry in 20 pairs of GC tumor tissues and adjacent non-tumor gastric tissues. Data are presented as the mean ± SD. *p < 0.05; **p < 0.01; ***p < 0.001: ****p < 0.0001

We also performed qRT-PCR to verify the reliability of DEGs calculated by bioinformatics methods. We found that these genes were highly expressed in most of GC cell lines and 20 pairs of GC tissue samples (Fig. 3A, B). This is consistent with our bioinformatics results. Meanwhile, western blot analysis revealed that the protein level of these 3 genes were also highly expressed in most of GC cell lines and tissue samples (Fig. 3C–F). And, the results of immunohistochemistry were consistent with the results of qRT-PCR and western blot (Fig. 3G, H). These results indicate that DPYS, NT5E and UPP1 are highly expressed in both RNA and protein levels in GC, which may be related to the formation and susceptibility of GC.

### Establishment of a prognostic risk model in TCGA

**Fig. 4:**
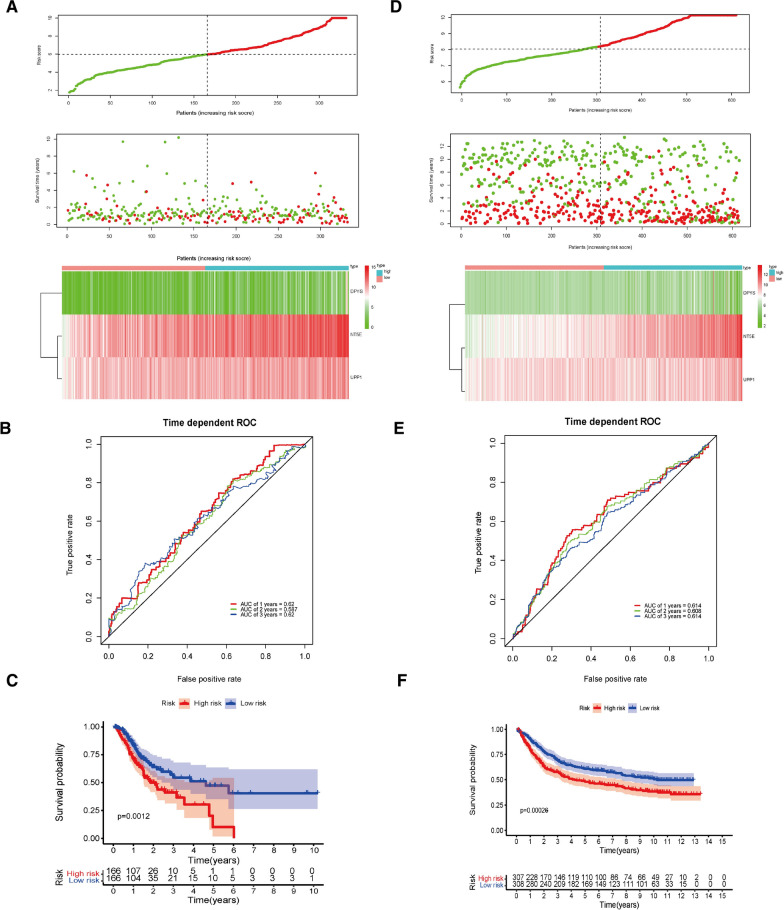
Risk score model, time-dependent ROC analysis, and survival analysis for the prognostic risk model. **A**–**C** Risk scoring model, time-dependent ROC analysis and survival analysis of genes related to pyrimidine metabolism in TCGA. **E**–**G** Risk scoring model, time-dependent ROC analysis and survival analysis of genes related to pyrimidine metabolism in GEO

On basis of the relationship between the mRNA expression levels of these three genes and the prognosis of GC, we established a prognostic risk model. The model was conducted as: prognostic risk score = 0.1661*NT5E + 0.1007*DPYS + 0.1877*UPP1. In this risk model, GC patients were segmented into high-risk cohort and low-risk cohort according to the optimal cutoff value of 6.30 (Fig. 4A). In the middle of Fig. 4A, we can see that patients in high-risk cohort had lower survival times than the low-risk cohort. The difference in expression of each gene between high-risk cohort and low-risk group was shown at the bottom of Fig. 4A and Additional file 2: Fig. S2A, all of these 3 genes are highly expressed in high-risk GC cohort. According to the ROC curve, the area under the curve (AUC) of OS in 1, 2, and 3 year were 0.62, 0.587 and 0.62, respectively, indicating that our model was relatively reliable (Fig. 4B). The OS in high-risk cohort was obviously lower than the low-risk cohort too (*p* = 0.0012, Fig. 4C). In conclusion, these results suggest that this prognostic risk model based on DEGs in pyrimidine metabolism can indeed guide the prognosis of GC patients.

### Validation of the prognostic risk model in GEO

We used clinical information from GSE15459 and GSE84433 as test databases to verify the validity of prognostic models for predicting GC outcomes. Similarly, GC patients were segmented into high-risk cohort and low-risk cohort with cutoff score at 8.0 (Fig. 4D, Additional file 2: Fig. S2B). According to the ROC curve, the AUC of OS in 1, 2, and 3 year were 0.614, 0.608, and 0.614, indicating that our model was quite reliable in DEO too (Fig. 4E). The results are consistent with those in TCGA, all of these 3 genes are highly expressed in GC, patients in high-risk cohort had lower survival times than the low-risk cohort in this model (Fig. 4D, F).

### The prognostic risk model is an important and independent clinical feature in GC

**Fig. 5:**
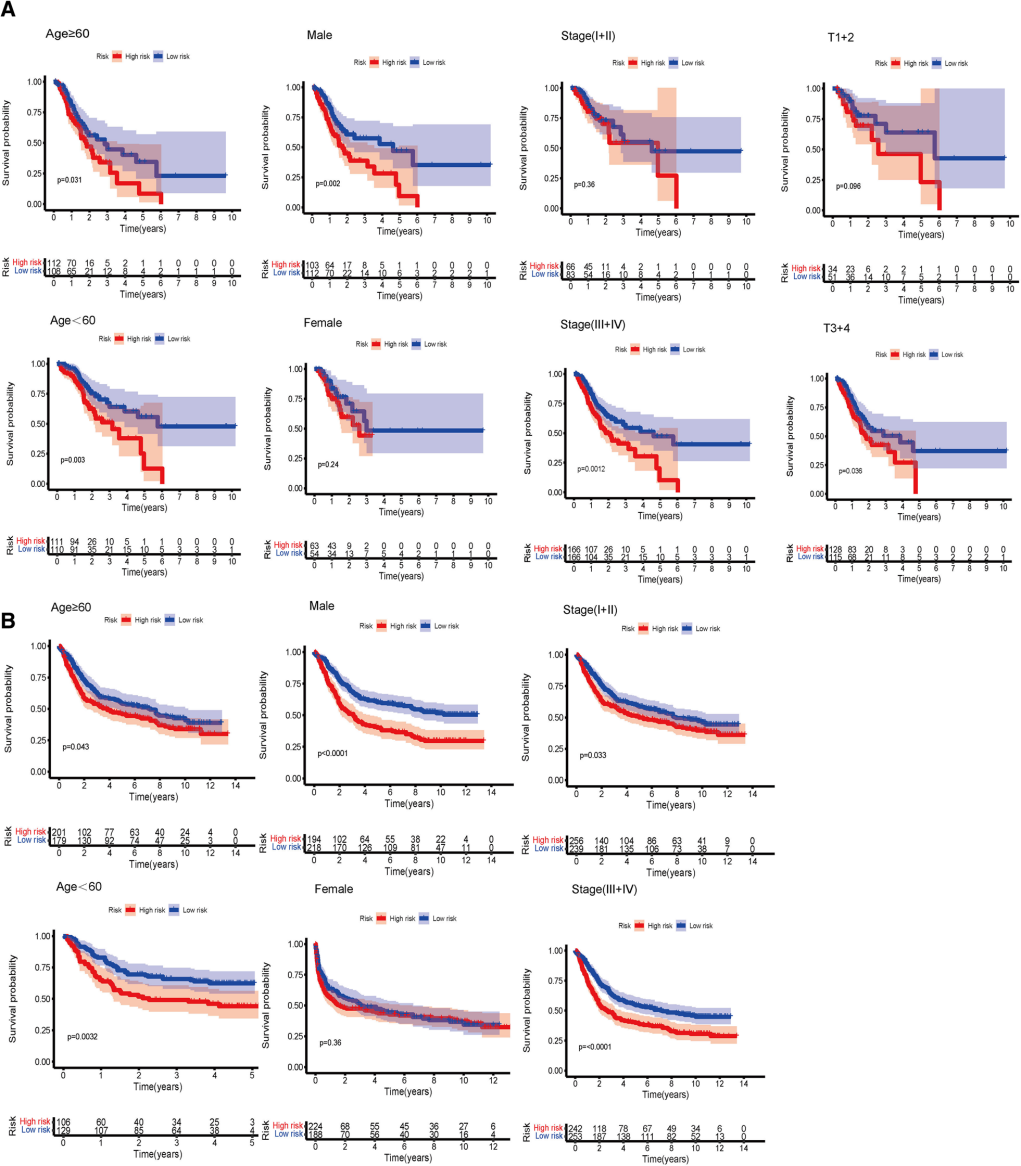
Stratified Kaplan–Meier curves of OS between high-risk group and low-risk group. **A** Kaplan–Meier curves of OS differences stratified by age, gender, tumor grade and TNM stage between high-risk group and low-risk group in TCGA. **B** Kaplan–Meier curves of OS differences stratified by age, gender, tumor grade and TNM stage between high-risk group and low-risk group in GEO

The OS was stratified according to general clinical features, and the difference between the low-risk cohort and the high-risk cohort was analyzed. According to subgroup classification including age, gender, tumor stage and grade, the OS in high-risk cohort was generally worse than that in low-risk cohort (Fig. 5A). Similar result can be obtained in the GEO database. However, since there is no grade information in GEO database, we could not perform stratified survival analysis for grade in GEO database (Fig. 5B).

**Fig. 6:**
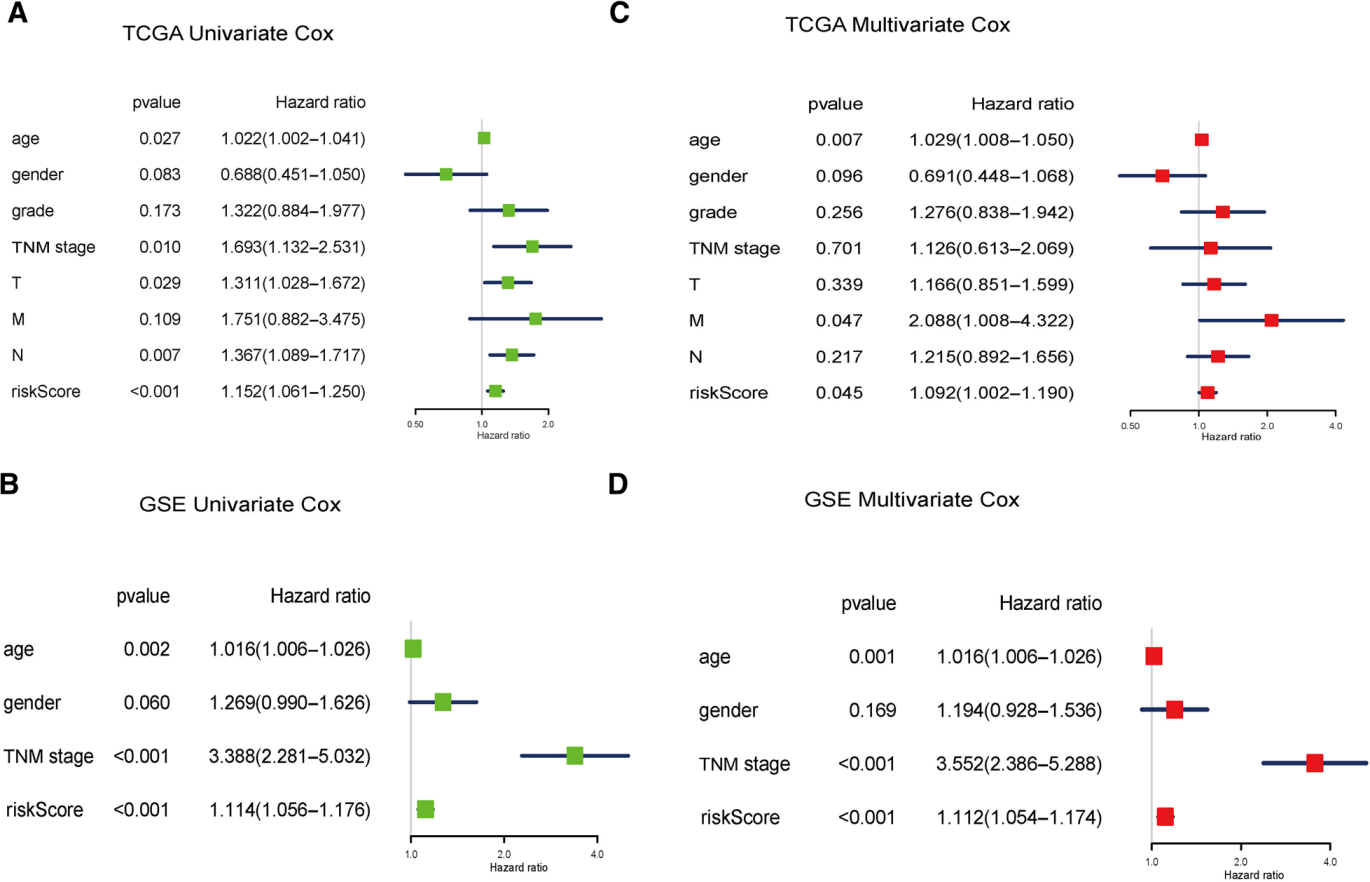
Univariate and multivariate analyses of factors associated with survival. **A** Univariate analysis of overall survival risk factors in pyrimidine metabolism in TCGA. **B** Univariate analysis of overall survival risk factors in pyrimidine metabolism in GEO. **C** Multivariate analysis of overall survival risk factors in pyrimidine metabolism in TCGA. **D** Multivariate analysis of overall survival risk factors in pyrimidine metabolism in GEO

Next, in order to judge whether the model is an important and independent clinical feature of GC prognosis, we carried out univariate and multivariate Cox regression analysis in TCGA and GEO respectively. In TCGA, the hazard ratio (HR) value of the risk score model was 1.152, and the 95% confidence interval (CI) was 1.061–1.250 in univariate analyses (*p* < 0.001) (Fig. 6A), suggesting that risk model was an important clinical feature. Similar conclusions were also verified in the GEO database, the hazard ratio (HR) value was 1.114, and the 95% CI was 1.056–1.176 (*p* < 0.001). It was the second most important clinical feature after TNM stage (Fig. 6B). In multivariate Cox regression analysis, the HR of this model based on pyrimidine metabolism was 1.092 and 95% CI was 1.002–1.190 (*p* = 0.045) in TCGA (Fig. 6C), and the HR of the risk model was 1.112 and 95% CI was 1.054–1.174 (*p* < 0.001) in GEO (Fig. 6D). These suggested that this model could be utilized as an independent clinical feature to judge the prognosis of GC. We have noticed that there are some differences between the results in TCGA and GEO databases. For example, the clinical factor TNM stage was not an independent factor in TCGA database, but it was indeed shown as an independent clinical factor in GEO database, which may be related to the difference of cases contained in the two databases.

### Establishment and validation of a prognostic nomogram

**Fig. 7:**
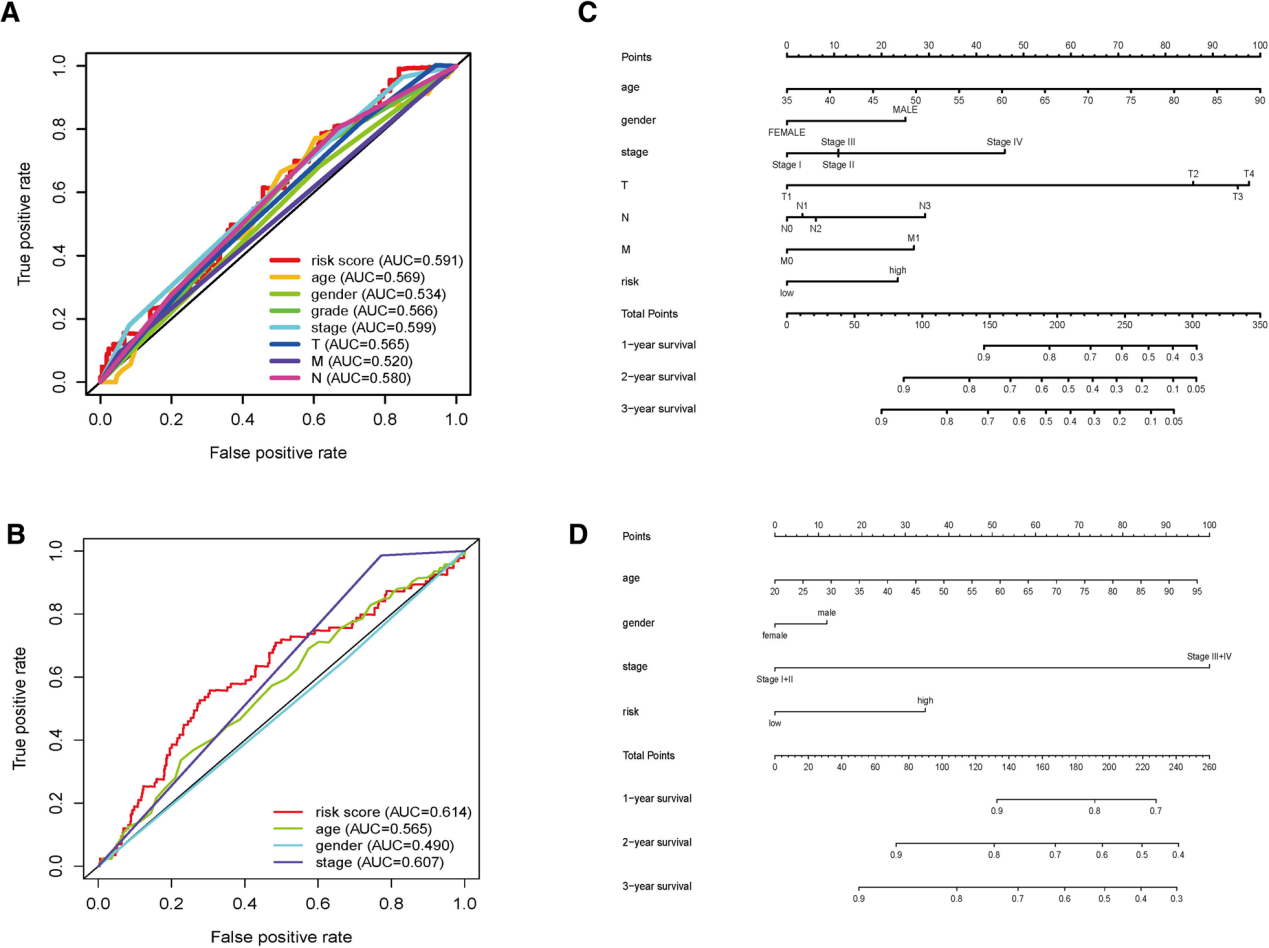
Establishment and validation of a prognostic nomogram. **A** ROC curves of clinical characters and risk score based on pyrimidine metabolism in TCGA. **B** ROC curves of clinical characters and risk score based on pyrimidine metabolism in GEO. **C** The nomogram predicts the probability of the 1, 2, 3 year OS related to pyrimidine metabolism in TCGA. **D** The nomogram predicts the probability of the 1, 2, 3 year OS related to pyrimidine metabolism in GEO

According to above analysis, we know that prognostic risk model is one of the important and independent clinical features that can guide the prognosis of GC. Here we carried out a multivariate Cox regression on this model and found that the AUC of this model was higher than other clinical indictors, showing that the prognostic risk model was comparatively dependable (Fig. 7A, B). Therefore, for purpose of intuitively describing the influence of various clinical factors on patients’ OS, including the prognostic risk model we established. We made nomograms to predict the incidence of OS at 1, 2, and 3 years in the TCGA and GEO databases, respectively (Fig. 7C, D).

### GSEA pathway analysis

To identify pathways that might be affected by pyrimidine metabolism disorders, GSEA pathway analysis was carried out. The five representative pathways enriched in the high-risk cohort were apoptosis, pathogenic Escherichia coli infection, pyrimidine metabolism, sphingolipid metabolism and vibrio cholerae infection (Additional file 3: Fig. S3A, B), the five representative pathways enriched in low-risk cohort were drug metabolism cytochrome p450, histidine metabolism, long term depression, metabolism of xenobiotics by cytochrome p450 and tryptophan metabolism, but the five representative pathways enriched in the low-risk cohort were not statistically significant in TCGA (Additional file 3: Fig. S3C, D).

## Discussion

In our study, we established a prognostic risk model based on three genes (NT5E, UPP1 and DPYS) found to be associated with GC pyrimidine metabolism and demonstrated that this prognostic risk model is a reliable and independent clinical feature of GC. We also performed GSEA pathway analysis to explore the pathways that may be affected by the disorder of pyrimidine metabolism. Finally, we made a nomogram to visually map the impact of the model and other important clinical measures on the OS of patients.

Although there have been reports that these three genes are more or less involved in cancer progression, we are the first to integrate all DEGs in pyrimidine metabolism. NT5E is a cell surface protein anchored by glycosylphosphatidylinositol [22, 23]. It is the first crucial enzyme in the purinergic signaling pathway [24, 25]. In recent years, purinergic signaling pathways with extracellular adenosine, AMP and ATP as the main signaling molecules have been found to play a significant part in the progression of some tumors, including GC [26]. NT5E overexpression was observed in GC tissues and serum, and it is connected to the clinical progression of GC patients [15, 16]. Overexpression of NT5E can promote tumor proliferation, migration and invasion [27, 28]. DPYS (also known as DHP) is a zinc metalloenzyme, which is highly expressed in tumors compared with the matching normal tissues, whose role is to degrade dihydropyrimidine [29]. Excessive accumulation of dihydropyrimidine will facilitate the constitution of DNA–protein crosslinks, leading to DNA replication and transcriptional stress [29]. Studies have shown that DPYS subtype DPYSL3 was a promising biomarker for GC malignant behavior [30]. UPP1 catalyzes the reversible phosphorylation of uridine or 2'- deoxyuridine to uracil and ribose-1-phosphate (or deoxyribose-1-phosphate), plays an essential role in pyrimidine recovery and uridine homeostasis regulation [31]. UPP1 as an oncogene has been revealed to be involved in numerous malignant tumors, for example, colorectal cancer and thyroid cancer, etc. [32] Researches have investigated the connections between the expression of UPP1 and the prognosis of cancer patients. They demonstrated that the higher the level of UPP1 in the tumor, the worse the prognosis and the shorter the survival time of cancer patients [33]. But genes are not an isolated island, the interactions between genes form a beautiful pulsating map of life. Although all three genes have been reported to be involved in the progression of GC, further experiments are needed to determine whether these genes can conjointly target GC or other types of cancer.

Our research also has many shortcomings. We noticed that except for NT5E, which is a key enzyme in purinergic signaling pathway, neither UPP1 nor DPYS are key enzymes in pyrimidine metabolism. According to previous reports, rate-limiting enzymes such as CAD and DHODH are essential in the progress of tumors. In addition to the selection of samples, the TCGA dataset lacks clinical information on the clinical variables associated with tumor progression and postoperatively, such as tumor size, vascular invasion, recurrence of GC, and postoperative treatment, which may also be influencing factors [34]. Therefore, our research cannot eliminate the survival of patients may be influenced by postoperative treatment or other key clinical features. And due to incomplete clinical data, we excluded some part of the TCGA cohort for further analysis, this may influence the accuracy of the statistics too. In addition, like all studies that have identified genes in other metabolic pathways as prognostic factors for GC [35, 36], since our prognostic risk model was constructed by us through the online database, its role in the current reality is not clear, which needs to be further confirmed by our subsequent experiments.

In conclusion, our research shows that pyrimidine metabolism is disturbed in GC, and predicts GC prognosis. The prognostic risk model composed of three pyrimidine metabolism genes (NT5E, UPP1 and DPYS) could be used as an important and independent biomarker for predicting GC prognosis.

## Supplementary Information

**Additional file 1: Figure S1.** Lasso analysis of genes related to pyrimidine metabolism in GC. (A-B) Lasso analysis of genes related to pyrimidine metabolism in GC.

**Additional file 2: Figure S2. **Difference in expression levels of these three DEGS between the high-risk group and the low-risk group. (A) In TCGA database. (B) In GEO database.

**Additional file 3: Figure S3. **The significantly enriched KEGG pathways by GESA. (A) Five representative KEGG pathways in the high-risk group of GC pyrimidine metabolism in TCGA. (B) Five representative KEGG pathways in the high-risk group of GC pyrimidine metabolism in GEO. (C) Five representative KEGG pathways in the low-risk group of GC pyrimidine metabolism in TCGA. (D) Five representative KEGG pathways in the low-risk group of GC pyrimidine metabolism in GEO.

## Data Availability

The data analyzed in this study were obtained from TCGA database, GSE15459 and GSE8443 in GEO database.

## Abbreviations

GC: Gastric cancer
DEGs: Differentially expressed genes
CAD: Carbamoyl-phosphate synthetase 2, aspartate transcarbamylase, and dihydroorotase
NT5C2: 5ʹ-Nucleotidase, cytosolic II
RRM1: Ribonucleotide reductase catalytic subunit M1
TK1: Thymidine kinase 1
DHODH: Dihydroorotate dehydrogenase
UCK2: Uridine-cytidine kinase 2
NT5E: Ecto-5-nucleotidase
5FU: 5-Fluorouracil
FPKM: Fragments per kilobase million
TCGA: The Cancer Genome Atlas
GEO: The Gene Expression Omnibus
ROC: Receiver operating characteristic
OS: Overall survival
GSEA: Gene set enrichment analyses
DPYS: Dihydropyrimidinase
UPP1: Uridine phosphorylase 1
PPI: Protein–protein interaction
AUC: Area under the curve
HR: Hazard ratio
CI: Confidence interval
