# Supplementary material for: Identification of crucial genes of pyrimidine metabolism as biomarkers for gastric cancer prognosis
Source: Cancer Cell Int. 2021 Dec 14;21:668. doi: 10.1186/s12935-021-02385-x (PMC8670209; doi:10.1186/s12935-021-02385-x)
Supplement: Supplementary file 1 — Additional file 1: Figure S1. Lasso analysis of genes related to pyrimidine metabolism in GC. (A-B) Lasso analysis of genes related to pyrimidine metabolism in GC. [file 12935_2021_2385_MOESM1_ESM.docx]

**
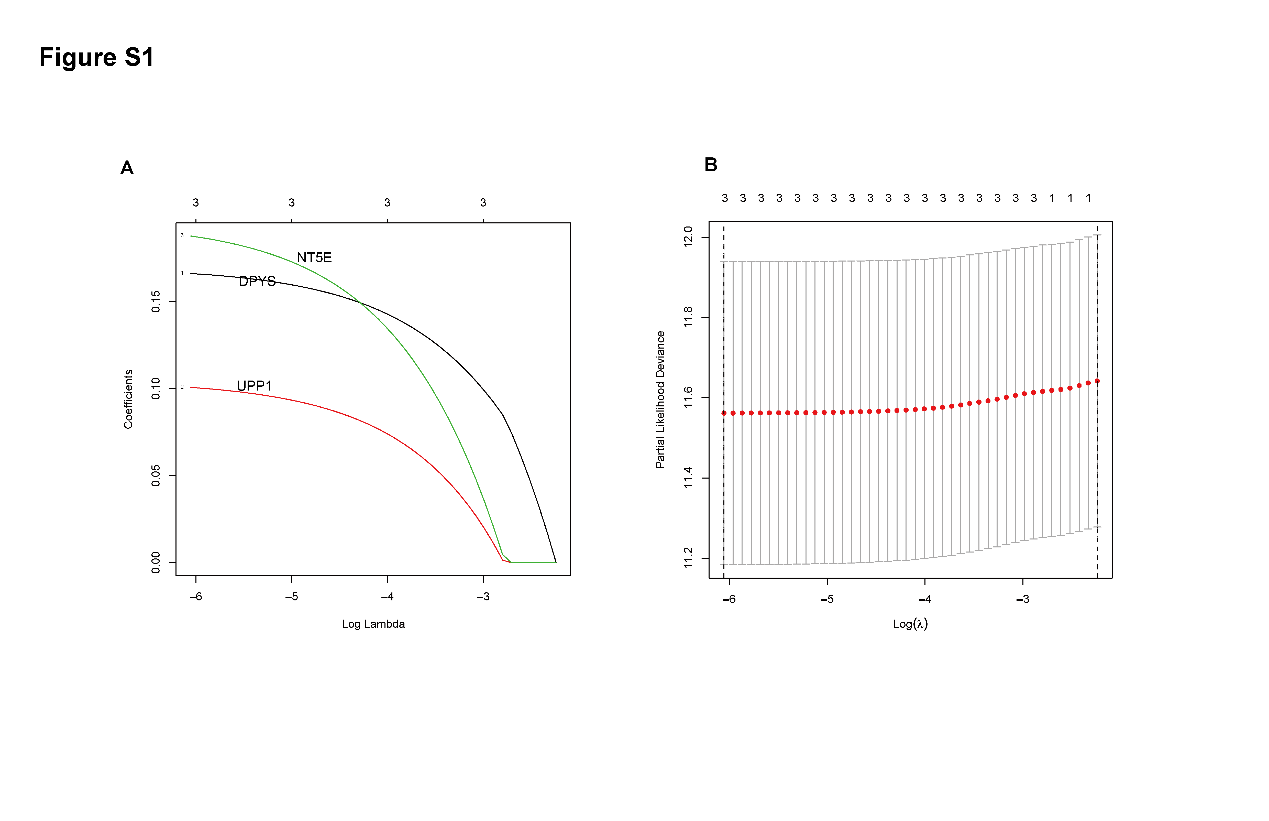
**

**Additional file 1: Figure S1. Lasso analysis of genes related to pyrimidine metabolism in GC.** (A-B) Lasso analysis of genes related to pyrimidine metabolism in GC.
