# Supplementary material for: Identification of crucial genes of pyrimidine metabolism as biomarkers for gastric cancer prognosis
Source: Cancer Cell Int. 2021 Dec 14;21:668. doi: 10.1186/s12935-021-02385-x (PMC8670209; doi:10.1186/s12935-021-02385-x)
Supplement: Supplementary file 2 — Additional file 2: Figure S2. Difference in expression levels of these three DEGS between the high-risk group and the low-risk group. (A) In TCGA database. (B) In GEO database. [file 12935_2021_2385_MOESM2_ESM.docx]

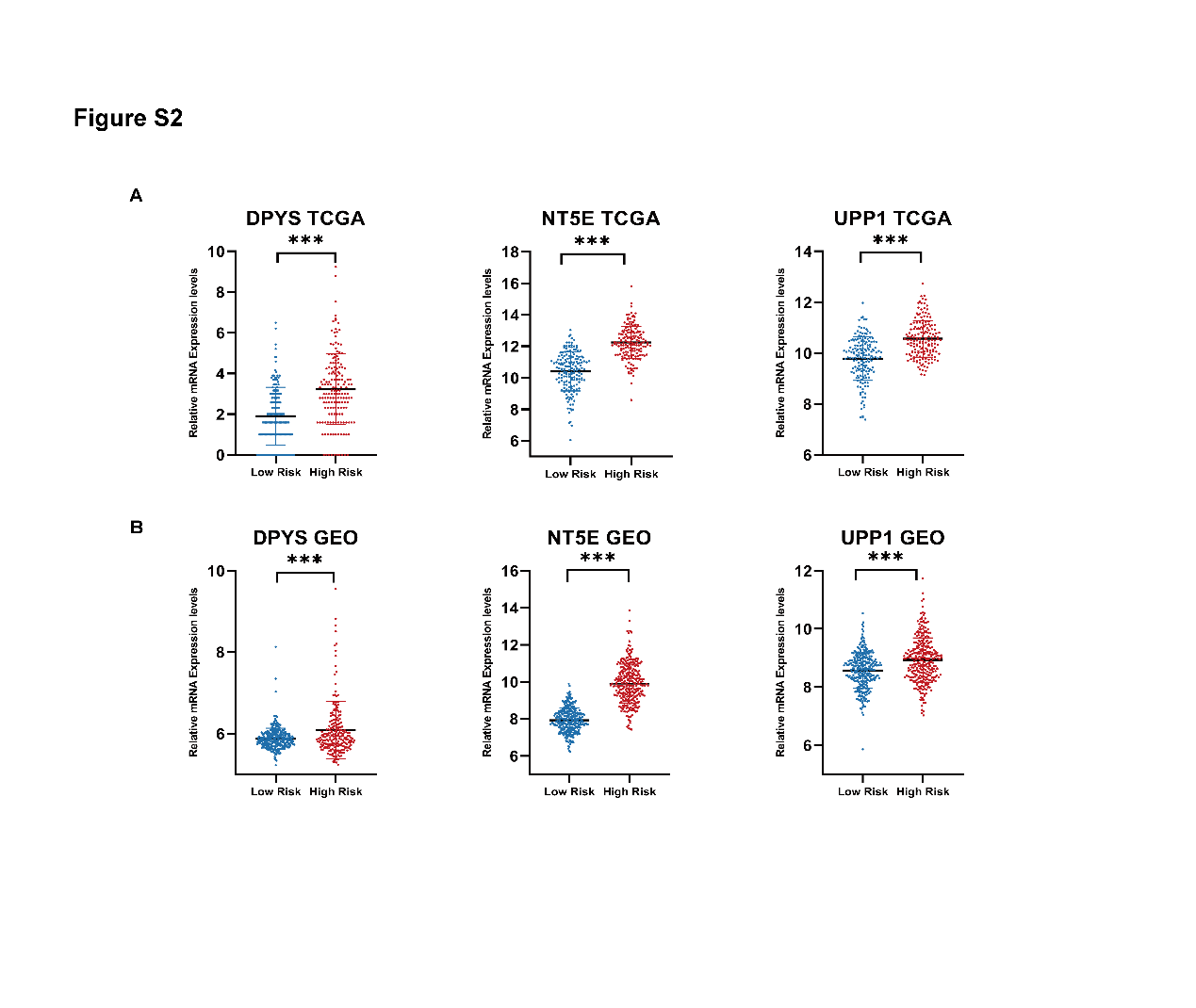


**Additional file 2: Figure S2. Difference in expression levels of these three DEGS between the high-risk group and the low-risk group.** (A) In TCGA database. (B) In GEO database.
