# Supplementary material for: Identification of crucial genes of pyrimidine metabolism as biomarkers for gastric cancer prognosis
Source: Cancer Cell Int. 2021 Dec 14;21:668. doi: 10.1186/s12935-021-02385-x (PMC8670209; doi:10.1186/s12935-021-02385-x)
Supplement: Supplementary file 3 — Additional file 3: Figure S3. The significantly enriched KEGG pathways by GESA. (A) Five representative KEGG pathways in the high-risk group of GC pyrimidine metabolism in TCGA. (B) Five representative KEGG pathways in the high-risk group of GC pyrimidine metabolism in GEO. (C) Five representative KEGG pathways in the low-risk group of GC pyrimidine metabolism in TCGA. (D) Five representative KEGG pathways in the low-risk group of GC pyrimidine metabolism in GEO. [file 12935_2021_2385_MOESM3_ESM.docx]

**Additiobnal file 3: Figure S3. The significantly enriched KEGG pathways by GESA.** (A) Five representative KEGG pathways in the high-risk group of GC pyrimidine metabolism in TCGA. (B) Five representative KEGG pathways in the high-risk group of GC pyrimidine metabolism in GEO. (C) Five representative KEGG pathways in the low-risk group of GC pyrimidine metabolism in TCGA. (D) Five representative KEGG pathways in the low-risk group of GC pyrimidine metabolism in GEO.
